# Supplementary material for: Phosphoregulation of RAD51AP1 function in homology-directed repair
Source: J Biol Chem. 2026 Jan 12;302(3):111149. doi: 10.1016/j.jbc.2026.111149 (PMC12887393; doi:10.1016/j.jbc.2026.111149)
Supplement: Supplementary Material — 2 [file mmc2.pdf]

Supporting Information

Phosphoregulation of RAD51AP1 function in homology-directed repair

Neelam Sharma [1], Mollie E. Uhrig [1,2], Youngho Kwon [3], Patrick Sung [3], Claudia Wiese [1*]

Supporting Figure S1
Supporting Figure S2
Supporting Figure S3
Supporting Figure S4
Supporting Figure S5

Supporting Table S1

Supporting Experimental Procedures

Supporting References

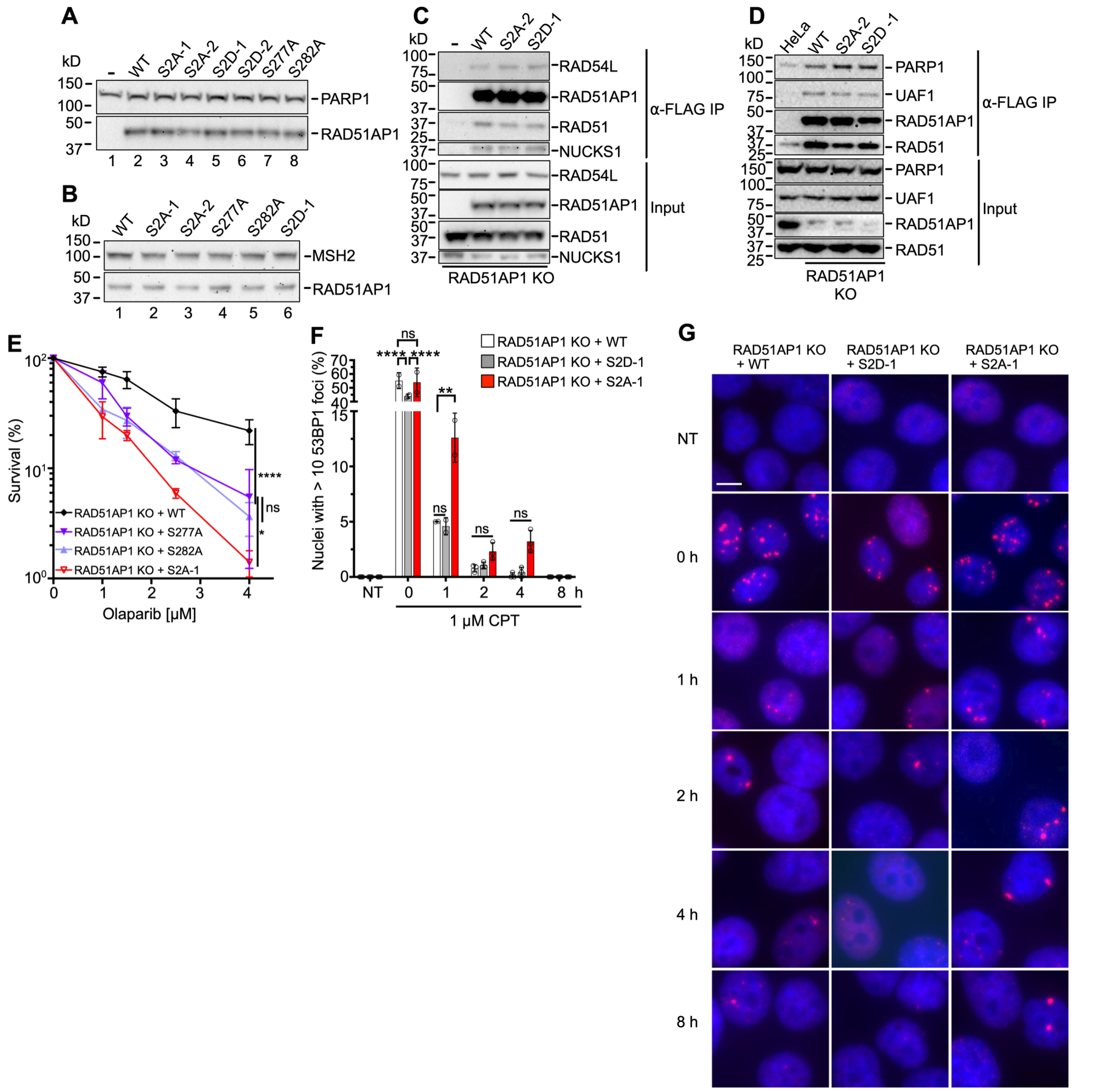

Figure S1. Related to Figure1. **RAD51AP1 variants retain the known RAD51AP1 protein interactions in cells.** *A-B*, Western blots to show the expression of ectopic RAD51AP1 and mutants in HeLa RAD51AP1 KO cells. For RAD51AP1-S2A and RAD51AP1-S2D, two independently isolated clones were analyzed. PARP1, MSH2: loading controls. *C-D*, Western blots after anti-FLAG co-immunoprecipitations to show that ectopic RAD51AP1 and RAD51AP1 mutants (-S2A and -S2D) retain the described protein interactions of endogenous RAD51AP1(1-4). *E*, Results from clonogenic cell survival assays in response to Olaparib to determine the sensitivity of cells expressing RAD51AP1 with single residue changes (S277A or S282A) in comparison to cells expressing RAD51AP1-S2A. Two-way ANOVA followed by Tukey's multiple comparison test. **p* < 0.05. *****p* < 0.0001. ns, not significant. *F*, Time course of 53BP1 foci formation after treatment of HeLa RAD51AP1 KO cells and derivatives with 1 μM CPT for 1 h (n = 3). NT: not treated. Data points are independent biological replicates. Bars are the means ± 1 SD. Two-way ANOVA followed by Tukey's multiple comparison test. ***p* < 0.01. *****p* < 0.0001. ns, not significant. *G*, Representative micrographs of 53BP1 foci (red) for the results shown in (F). DAPI: blue. Scale bar: 10 μm.

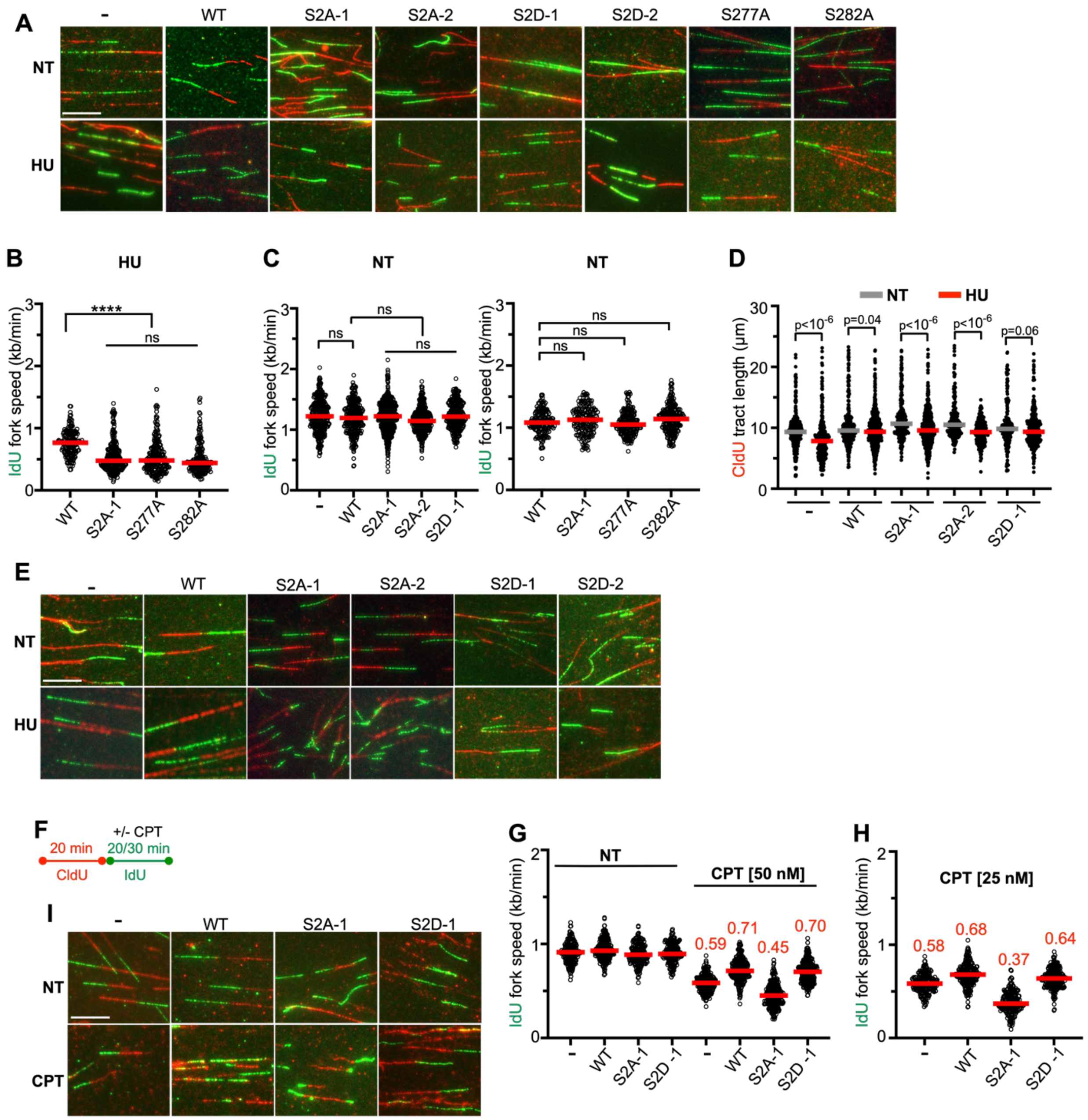

Figure S2. Related to Figure 2. **RAD51AP1-S2A, -S277A, and -S282A cells exhibit impaired replication fork restart and fork progression in response to replication stress.** *A*, Representative micrographs of DNA fibers in HeLa RAD51AP1 KO cells and derivatives under unperturbed conditions (NT) and after HU to determine recovery from stalled replication by IdU fork progression (green; Fig. 2A-C) and fork resection by CldU fork progression (red; Fig. 2*A*, S2*D*). *B*, Dot plot with medians of IdU fork speeds in HeLa RAD51AP1 KO cells expressing RAD51AP1 or mutants to show fork restart after HU (labeling protocol shown in Fig. 2A; n = 3;100-150 fiber tracts/experiment analyzed). ****$p < 0.0001$. ns, not significant. *C*, Dot plot with medians of IdU fork speeds in unperturbed HeLa RAD51AP1 KO cells (-) and derivatives (n = 3; 100-150 fiber tracts/experiment analyzed). ns, not significant. Kruskal-Wallis test followed by Dunn's multiple comparisons test. *D*, Dot plot with medians of CldU tract lengths in unperturbed and HU treated HeLa RAD51AP1 KO cells (-) and derivatives (n=3 for -, WT, S2A-1, S2D-1 cells; n = 2 for S2A-2 cells; 100-150 fiber tracts/experiment analyzed). Mann-Whitney test. *E*,

Representative micrographs of DNA fibers in HeLa RAD51AP1 KO cells (-) and derivatives under unperturbed conditions (NT) and after mild replication stress (labeling protocol shown in Fig. 2D). *F*, Schematic for the protocol of the DNA fiber assay to determine fork progression in the presence of mild replication stress by camptothecin (CPT) and used in (G-I). *G*, Dot plot with medians of IdU fork speeds in HeLa RAD51AP1 KO cells (-) and derivatives without (NT) and in the presence of 50 nM CPT (n = 1; 150-200 fiber tracts analyzed). *H*, Dot plot with medians of IdU fork speeds in HeLa RAD51AP1 KO cells (-) and derivatives in the presence of 25 nM CPT (n = 1; 150-200 fiber tracts analyzed). *I*, Representative micrographs of DNA fibers in HeLa RAD51AP1 KO cells (-) and derivatives under unperturbed conditions (NT) and after mild replication stress (25 nM CPT). Scale bars: 10 µm.

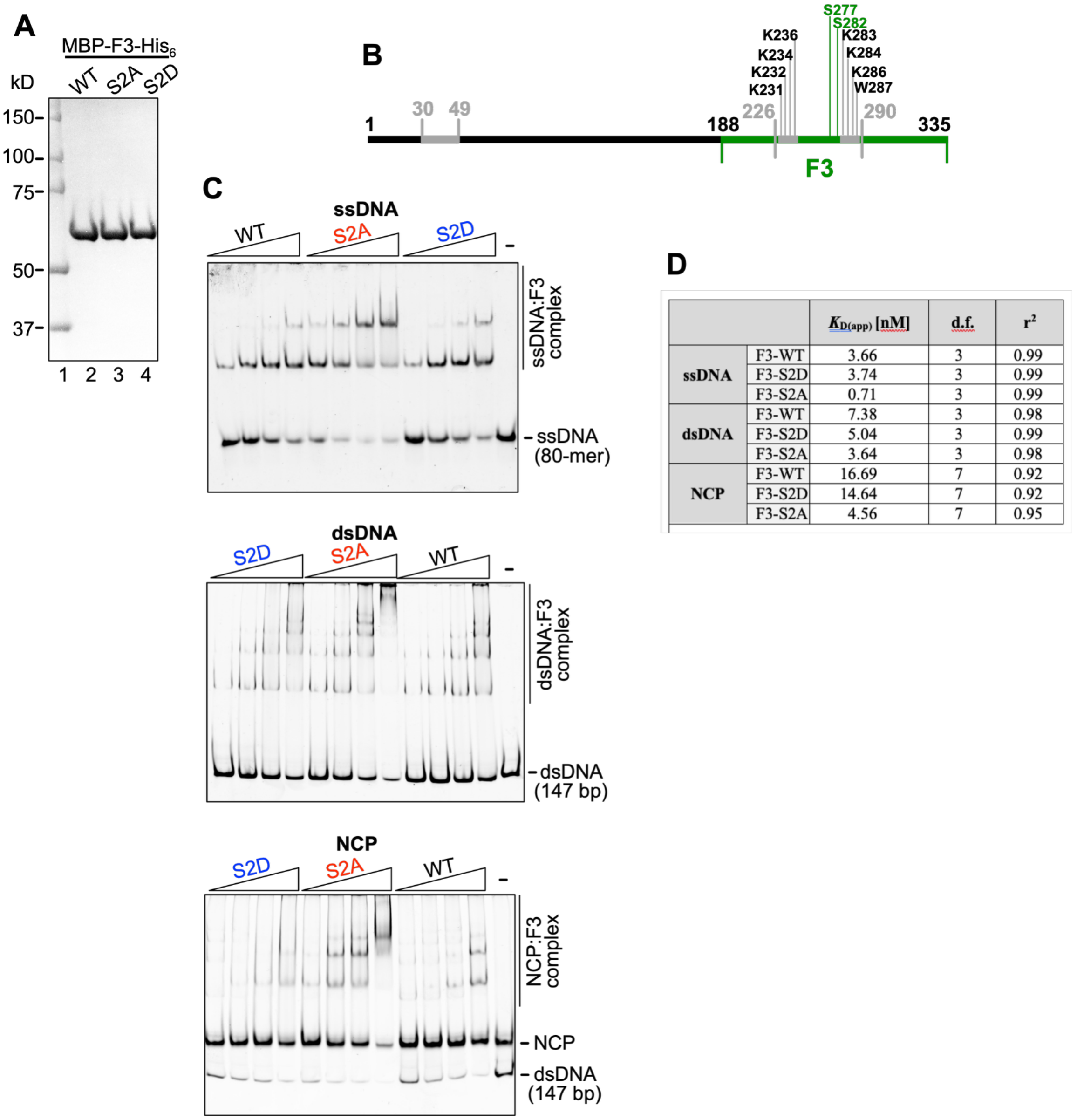

		$K_{D(app)}$ [nM]	d.f.	r^2
ssDNA	F3-WT	3.66	3	0.99
	F3-S2D	3.74	3	0.99
	F3-S2A	0.71	3	0.99
dsDNA	F3-WT	7.38	3	0.98
	F3-S2D	5.04	3	0.99
	F3-S2A	3.64	3	0.98
NCP	F3-WT	16.69	7	0.92
	F3-S2D	14.64	7	0.92
	F3-S2A	4.56	7	0.95

Figure S3. Related to Figure 3. **S277/282 regulate RAD51AP1 binding to ssDNA, dsDNA, and the NCP.** *A*, SDS-PAGE to show purified MBP-F3-His6 and mutants (1 µg protein each). *B*, Schematic representation of the RAD51AP1 protein (isoform 2; *black*) and the RAD51AP1-F3 fragment (residues 188-335; *green*) containing the bipartite DNA binding domain (grey) and the critical residues engaged in DNA binding and previously identified in this domain (5). Location of residues S277 and S282 is indicated. *C*, Representative EMSAs showing the mobility shifts for

MBP-F3-His6 and mutants with ssDNA, dsDNA, and the NCP. *D*, Summary of apparent K_D values determined by fitting the binding data to a one-site specific binding model using non-linear regression in GraphPad Prism 10.

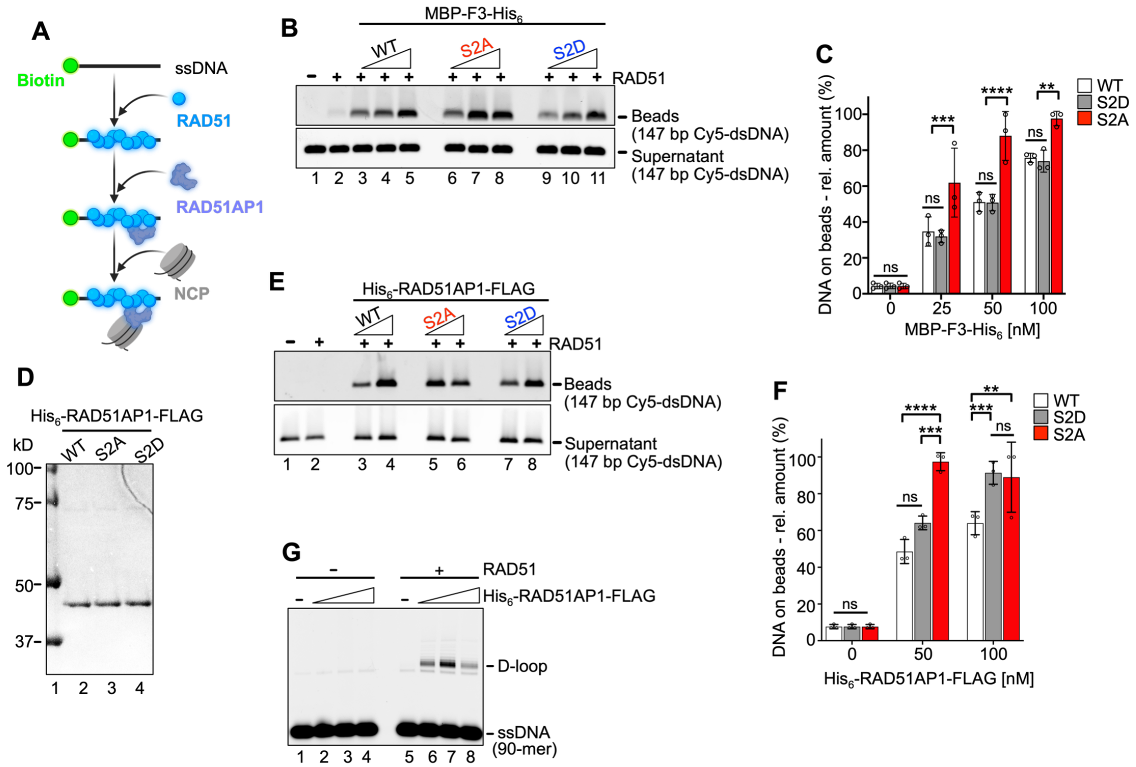

Figure S4. Related to Figure 4. **S277/282 regulate RAD51AP1's stimulation of RAD51 in duplex capture with the NCP.** *A*, Schematic of the duplex capture assay with His6-RAD51AP1-FLAG (or mutants) and the NCP. *B*, Representative agarose gel to quantify duplex capture (*i.e.*, DNA on beads) with MBP-F3-His6 or mutants and the NCP. *C*, Quantitation of duplex capture with MBP-F3-His6 or mutants (n = 3). Data points are independent biological replicates. Bars are the means ± 1 SD. *D*, SDS-PAGE to show purified His6-RAD51AP1-FLAG and mutants (250 ng protein each). *E*, Representative agarose gel to quantify duplex capture (*i.e.*, DNA on beads) with full-length His6-RAD51AP1-FLAG or mutants and the NCP. *F*, Quantitation of duplex capture with full-length His6-RAD51AP1-FLAG or mutants (n = 3). Data points are independent biological replicates. Bars are the means ± 1 SD. *G*, Agarose gel to show that the stimulation of D-loop formation by RAD51AP1 (50-200 nM) is dependent on RAD51 (lanes 6-8). Two-way ANOVA followed by Tukey's multiple comparison test. $**p < 0.01$. $***p < 0.001$. $****p < 0.0001$. ns, not significant.

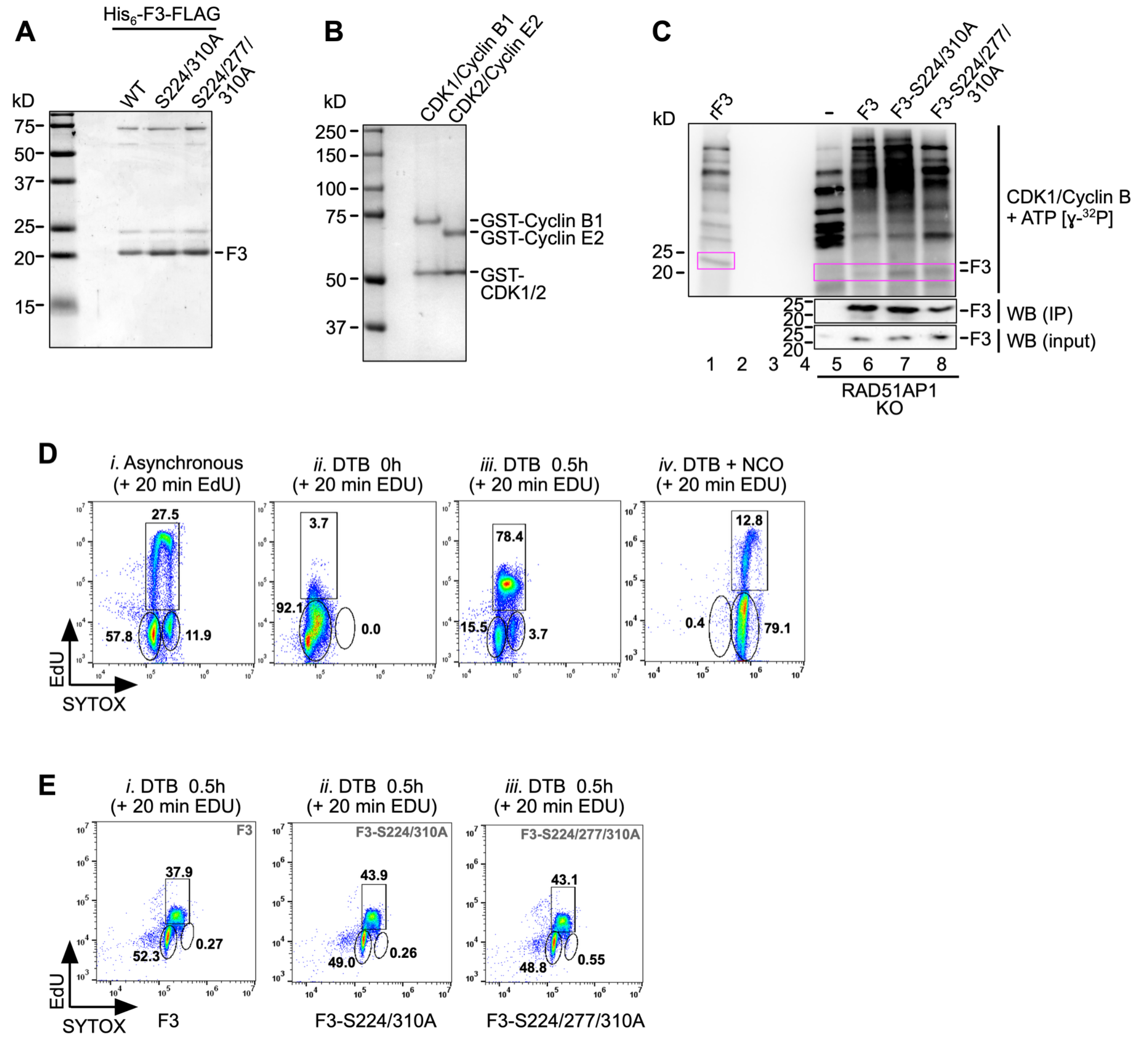

Figure S5. Related to Figure 5. **S277 is a CDK2 target in cells.** *A*, SDS-PAGE to show purified His_6-F3-FLAG and mutants (250 ng protein each). *B*, SDS-PAGE to show commercially obtained CDK1/Cyclin B1 (Abcam; ab271456) and CDK2/Cyclin E2 complexes (Abcam; ab268396) (500 ng protein complex each). *C*, Results from kinase reaction of transiently expressed (in HeLa RAD51AP1 KO cells) and precipitated FLAG-F3 mutants (S224/310A and S224/277/310A) with CDK1/Cyclin B1 and ɣ-^{32}P-ATP. Upper: Phosphorimage; middle and lower: Western blot with α-FLAG antibody. The product of the kinase reaction with purified (from *E. coli*) His_6-F3-FLAG is shown for comparison purposes (rF3, lane 1). *D*, Representative results from flow cytometry showing two-color fluorescence of cell cycle profiles of asynchronous RAD51AP1 KO cells stably expressing wild-type RAD51AP1, and of these cells synchronized by a DTB and released into regular growth medium without and with NCO. *E*, Representative results from flow cytometry showing two-color fluorescence of cell cycle profiles of RAD51AP1 KO cells transiently transfected to express F3, F3-S224/310A, or F3-S224/277/310A and released into regular growth medium (for 0.5 h) after a DTB. Prior to fixation, cells were pulse-labeled by EdU. Y-axis: EdU, X-axis: SYTOX.

Table S1. List of oligonucleotides/primers used in this study[1].

Template/ plasmid	Oligonucleotide	Sequence (5´-3´)
FLAG-RAD51AP1 into pENTR1A (from pOK24 (1))	5´-FLAG forward	ATCGGTCGACCCACCATGGATTACAAAGACGATGACGATAAAGGTGTGCGGCCTGTGAGACATAAGAAACC
	reverse	ATCGGTACCTCAGGTGCTAGTGGCATTTGGATGCAAAG
FLAG-RAD51AP1-S282A (in pENTR1A)	S282A forward	TCAGCTGAAgcaAAGAAACCTAAATGG
	S282A reverse	AGGGTCGCGTATTTCTAATG
FLAG-RAD51AP1-S277/282A (in pENTR1A)	S277A forward	AGAAATACGCgcaCCTTCAGCTG
	S277A reverse	AATGGTTTCCTAGTGGTATC
FLAG-RAD51AP1-S282D (in pENTR1A)	S282D forward	GCTGAAgacAAGAAACCTAAATGGGTC
	S282D reverse	TGAAGGGTCGCGTATTTCTAATGGTTTC
FLAG-RAD51AP1-S277/282D (in pENTR1A)	S277D forward	GctgAAAGCAAGAAACCTAAATGGGTC
	S277D reverse	TGAAGGGATCGCGTATTTCTAATGGTTTC
His$_6$-RAD51AP1-S282A-FLAG (in His$_6$-RAD51AP1-FLAG /pQE-80L (6))	S282A forward	TCAGCTGAAgcaAAGAAACCTAAATGG
	S282A reverse	AGGGTCGCGTATTTCTAATG
His$_6$-RAD51AP1-S277/282A-FLAG (in His$_6$-RAD51AP1-FLAG/ pQE-80L (6))	S277A forward	AGAAATACGCgcaCCTTCAGCTG
	S277A reverse	AATGGTTTCCTAGTGGTATC
His$_6$-RAD51AP1-S282D-FLAG (in His$_6$-RAD51AP1-FLAG/ pQE-80L (6))	S282D forward	GCTGAAgacAAGAAACCTAAATGGGTC
	S282D reverse	TGAAGGGTCGCGTATTTCTAATGGTTTC
His$_6$-RAD51AP1-S277/282D-FLAG (in His$_6$-RAD51AP1-FLAG/ pQE-80L (6))	S277D forward	GctgAAAGCAAGAAACCTAAATGGGTC
	S277D reverse	TGAAGGGATCGCGTATTTCTAATGGTTTC

MBP-F3-S282A-His$_6$ (in MBP-F3-His$_6$/ pET24a (7))	S282A forward	TCAGCTGAAgcaAAGAAACCTAAATGG
	S282A reverse	AGGGTCGCGTATTTCTAATG
MBP-F3-S277/282A-His$_6$ (in MBP-F3-His$_6$/ pET24a (7))	S277A forward	AGAAATACGCgcaCCTTCAGCTG
	S277A reverse	AATGGTTTCCTAGTGGTATC
MBP-F3-S282D-His$_6$ (in MBP-F3-His$_6$/ pET24a (7))	S282D forward	GCTGAAgacAAGAAACCTAAATGGGTC
	S282D reverse	TGAAGGGTCGCGTATTTCTAATGGTTTC
MBP-F3-S277/282A-His$_6$ (in MBP-F3-His$_6$/ pET24a (7))	S277D forward	GgctGAAGACAAGAAACCTAAATGGGTC
	S277D reverse	TGAAGGATCGCGTATTTCTAATGGTTTC
His$_6$-F3-FLAG (from His$_6$-RAD51AP1-FLAG/pQE-80L (6))	F3 forward	ATCGGGATCCGATTCTGAGGATGATTCTG
	F3 reverse	CGATGTCGACTCATTTATCGTCATCGTCTTTGTAA TCACCGGTGCTAGTGGCATTTGGATGCAAAGG
His$_6$-F3-S310A-FLAG (in pQE-80L)	S310A forward	AGAAATACGCgcaCCTTCAGCTG
	S310A reverse	AATGGTTTCCTAGTGGTATC
His$_6$-F3-S224/310A-FLAG (in pQE-80L)	S224A forward	GAAGGTAAAAgccCCAGTAGAAAAG
	S224A reverse	ACTTCTTTCTTTTTAATTTCTTTAACTTTAC
His$_6$-F3-S224/277/310A-FLAG (in pQE-80L)	S277A forward	AGAAATACGCgcaCCTTCAGCTG
	S277A reverse	AATGGTTTCCTAGTGGTATC
FLAG-F3 in pcDNA3.1 (from His6-F3-FLAG/pQE-80L)	forward	ATCGGCGGCCGCCCACCATGGATTACAAAGACGA TGACGATAAAGGTGATTCTGAGGATGATTCTGAT TTTTGTGAG
	reverse	CGATGGATCCTCAGGTGCTAGTGGCATTTGGATG CAAAG
Cy5-147 bp (from pUC19-601; (8))	5´Cy5-147bp forward	ATATCTGAGAATCCGGTGCCG
	147bp reverse	GGATGTATATATCTGACACGTGCCTG
biotin-ssDNA	80-mer	TCGTAGACAGCTCTAGCACCGCTTAAACGCACGT AGGCGCTGTCCCCCGCGTTTTAACCGCCAAGGGG ATTACTCCCTAG
Cy5-ssDNA	80-mer	TCGTAGACAGCTCTAGCACCGCTTAAACGCACGT AGGCGCTGTCCCCCGCGTTTTAACCGCCAAGGGG ATTACTCCCTAG
Cy5-ssDNA	90-mer	AAATCAATCTAAAGTATATATGAGTAAACTTGGT CTGACAGTTACCAATGCTTAATCAGTGAGGCACC TATCTCAGCGATCTGTCTATTT

[1]Mutated bases in lower case character.

Supporting Experimental Procedures

Duplex Capture Assay - The duplex capture assay was performed essentially as previously described with some modifications (9, 10). Briefly, the presynaptic filament was formed by incubating 5 µl of streptavidin-coated magnetic resin (Roche Molecular Biochemicals) with 5′-biotinylated 80-mer ssDNA oligonucleotide (5 µM; Table S1) and RAD51 (700 nM) in buffer A (25 mM Hepes, pH 7.5, 50 mM Tris-HCl, pH 7.5, 35 mM NaCl, 45 mM KCl, 1 mM $MgCl_2$, 0.16 mM EDTA, 2 mM ATP, 2% glycerol, 0.01% NP40, 0.4 mM ß-mercaptoethanol, and 100 µg/ml BSA) at 37°C for 5 min. The resin was captured magnetically and washed once with 20 µl buffer A. The wash was removed, and resin was resuspended in 10 µl buffer B (25 mM Tris-HCl, pH 7.5, 100 mM NaCl, 1.5 mM $MgCl_2$, and 1 mM DTT) containing His_6-RAD51AP1-FLAG (50 and100 nM) or MBP-F3-His_6 (25, 50, and 100 nM). After a 5-min incubation at 37°C, the resin was captured and washed. The supernatant was removed, and the resin was further incubated with 10 µl buffer A containing 1 µM NCP (with 147 bp Cy5-dsDNA) at 37°C for 10 min. The resin was captured, the supernatant was saved, and the resin was washed 4× with 200 µl buffer A. Both resin and supernatant were treated with 2 mg/ml Proteinase K in 2 mM Tris-HCl, pH 7.5, 1 mM $CaCl_2$, 0.2% SDS at 37°C for 15 min. Supernatant and resin-bound DNA were analyzed by 1% agarose gel electrophoresis. Image acquisition occurred on a Typhoon biomolecular imager (Cytiva). Quantification of signal intensities was done by ImageJ.

Supporting References

1. Kovalenko, O. V., Golub, E. I., Bray-Ward, P., Ward, D. C., and Radding, C. M. (1997) A novel nucleic acid-binding protein that interacts with human rad51 recombinase *Nucleic Acids Res* **25**, 4946–4953,
2. Liang, F., Longerich, S., Miller, A. S., Tang, C., Buzovetsky, O., Xiong, Y. *et al.* (2016) Promotion of RAD51-Mediated Homologous DNA Pairing by the RAD51AP1-UAF1 Complex *Cell reports* **15**, 2118–2126, 10.1016/j.celrep.2016.05.007
3. Selemenakis, P., Sharma, N., Uhrig, M. E., Katz, J., Kwon, Y., Sung, P. *et al.* (2022) RAD51AP1 and RAD54L Can Underpin Two Distinct RAD51-Dependent Routes of DNA Damage Repair via Homologous Recombination *Front Cell Dev Biol* **10**, 866601, 10.3389/fcell.2022.866601
4. Wiese, C., Dray, E., Groesser, T., San Filippo, J., Shi, I., Collins, D. W. *et al.* (2007) Promotion of homologous recombination and genomic stability by RAD51AP1 via RAD51 recombinase enhancement *Molecular cell* **28**, 482–490, S1097-2765(07)00597-7 [pii] 10.1016/j.molcel.2007.08.027
5. Dunlop, M. H., Dray, E., Zhao, W., San Filippo, J., Tsai, M. S., Leung, S. G. *et al.* (2012) Mechanistic insights into RAD51-associated protein 1 (RAD51AP1) action in homologous DNA repair *The Journal of biological chemistry* **287**, 12343–12347, 10.1074/jbc.C112.352161
6. Maranon, D. G., Sharma, N., Huang, Y., Selemenakis, P., Wang, M., Altina, N. *et al.* (2020) NUCKS1 promotes RAD54 activity in homologous recombination DNA repair *J Cell Biol* **219**, 10.1083/jcb.201911049
7. Dunlop, M. H., Dray, E., Zhao, W., Tsai, M. S., Wiese, C., Schild, D. *et al.* (2011) RAD51-associated protein 1 (RAD51AP1) interacts with the meiotic recombinase DMC1 through a conserved motif *J Biol Chem* **286**, 37328–37334, M111.290015 [pii] 10.1074/jbc.M111.290015
8. Lowary, P. T., and Widom, J. (1998) New DNA sequence rules for high affinity binding to histone octamer and sequence-directed nucleosome positioning *J Mol Biol* **276**, 19–42, 10.1006/jmbi.1997.1494

9. Pires, E., Sharma, N., Selemenakis, P., Wu, B., Huang, Y., Alimbetov, D. S. *et al.* (2021) RAD51AP1 mediates RAD51 activity through nucleosome interaction *J Biol Chem* 10.1016/j.jbc.2021.100844100844, 10.1016/j.jbc.2021.100844
10. Kobayashi, W., Takaku, M., Machida, S., Tachiwana, H., Maehara, K., Ohkawa, Y. *et al.* (2016) Chromatin architecture may dictate the target site for DMC1, but not for RAD51, during homologous pairing *Scientific reports* **6**, 24228, 10.1038/srep24228
